# One-year unplanned readmission after total hip arthroplasty in patients with osteonecrosis of the femoral head: rate, causes, and risk factors

**DOI:** 10.1186/s12891-023-06968-9

**Published:** 2023-10-26

**Authors:** Tianyu Wang, Congliang Gao, Dongwei Wu, Chengsi Li, Xinqun Cheng, Zhenbang Yang, Yingze Zhang, Yanbin Zhu

**Affiliations:** 1 Department of Orthopaedics, the 3rd Hospital, Hebei Medical University, NO.139 Ziqiang Road, Shijiazhuang, 050051 P.R. China; 2 Department of Orthopaedic Surgery, Huai’an Hospital of Huai’an City, Huai’an, Jiangsu 223200 P.R. China

**Keywords:** Osteonecrosis of the femoral head, Total hip arthroplasty, Unplanned readmission, Risk factors

## Abstract

**Background:**

The primary objectives of this study were to focus on one - year unplanned readmissions after THA in ONFH patients and to investigate rates, causes, and independent risk factors.

**Methods:**

Between October 2014 and April 2019, eligible patients undergoing THA were enrolled and divided into unplanned readmission within one year and no readmission in this study. All unplanned readmissions within 1 year of discharge were reviewed for causes and the rate of unplanned readmissions was calculated. Demographic information, ONFH characteristics, and treatment-related variables of both groups were compared and analysed.

**Results:**

Finally, 41 out of 876 patients experienced unplanned readmission. The readmission rate was 1.83% in 30 days 2.63% in 90 days, and 4.68% in 1 year. Prosthesis dislocation was always the most common cause at all time points studied within a year. The final logistic regression model revealed that higher risks of unplanned readmission were associated with age > 60 years (P = 0.001), urban residence (P = 0.001), ARCO stage IV (P = 0.025), and smoking (P = 0.033).

**Conclusions:**

We recommend the introduction of a strict smoking cessation program prior to surgery and the development of comprehensive management strategies, especially for the elderly and end-stage ONFH patients, and pay more attention to preventing prosthesis dislocation in the early days after surgery.

## Background

Osteonecrosis of the femoral head (ONFH) patients end up with the occurrence of subchondral collapse, severe destruction of the hip joint and loss of mobility, which places huge physical, mental, and financial burdens on family and society [1, 2]. Total hip arthroplasty (THA) is widely accepted as the most effective treatment for relieving pain and restoring mobility in the collasped stages [3, 4]. Unfortunately, compared to other diseases requiring THA, ONFH patients are potentially at higher risk of unplanned readmission [5, 6]. Unplanned readmission after THA is an adverse event with secondary injury, possible revision and even death, threatening patient health seriously [7, 8]. Meanwhile, this unwanted event also places a huge economic burden on patients and the healthcare system. According to statistics, the direct cost of each readmission after THA exceeds $ 17,000 [9]. Due to its severe harm, unplanned readmission has been included as a standard complication after THA proposed by the Hip Society in 2015 [10]. Some randomized prospective trials have proved that 12–75% of readmissions can be prevented [11]. Considering the huge and growing population of ONFH patients, and the predicted increase in THA utilization, more attention to this unplanned readmission is essential [11, 12].

Currently, although some studies have reported readmission after THA, none of them are conducted specifically in ONFH patients. Only fragmentary information is available. The reported readmission rates after THA in ONFH patients were considerable, ranging from 1.6 to 6.11% in 30 days and 9.6–13.1% in 90 day [5, 6, 13–15]. In a controlled study of ONFH and non-ONFH patients, Stavrakis et al. speculated that the high incidence of readmission in ONFH patients may be related to overall health and coexisting conditions associated with ONFH. It is regretful that they did not further analyze these specific readmission events [14]. In an observational cohort study, Singh et al. assessed the five most common causes of 90-day readmission in 2271 ONFH patients after THA, including prosthesis dislocation, unspecified septicemia, pneumonia, blood disease, and chronic lung disease [6]. However, they admitted that the study population lacked representativeness and some causes may be underestimated. To our best knowledge, none of existing studies have studied risk factors of unplanned readmission in ONFH patients. In addition, many previous studies can only report 30-day or 90-day outcomes, while a large number of readmission events often occur beyond this time.

Given the weakness and incompleteness of previous studies, further studies investigating this special readmission were urgently needed. Therefore, the main objectives of this study were to focus on readmissions after THA within one year in ONFH patients and to investigate its rates, causes, and independent risk factors.

## Methods

### Ethical issue

This study was approved by the Ethics Committee of the Third Hospital of Hebei Medical University. All procedures were performed according to the principles of the Declaration of Helsinki and in accordance with the guidelines of Strengthening the Reporting of Surgical Cohort Studies (STROCSS). All subjects were informed and consented. And to protect the privacy of patients, all data were anonymized by removing sensitive personal information.

### Patients and data collection

**Fig. 1:**
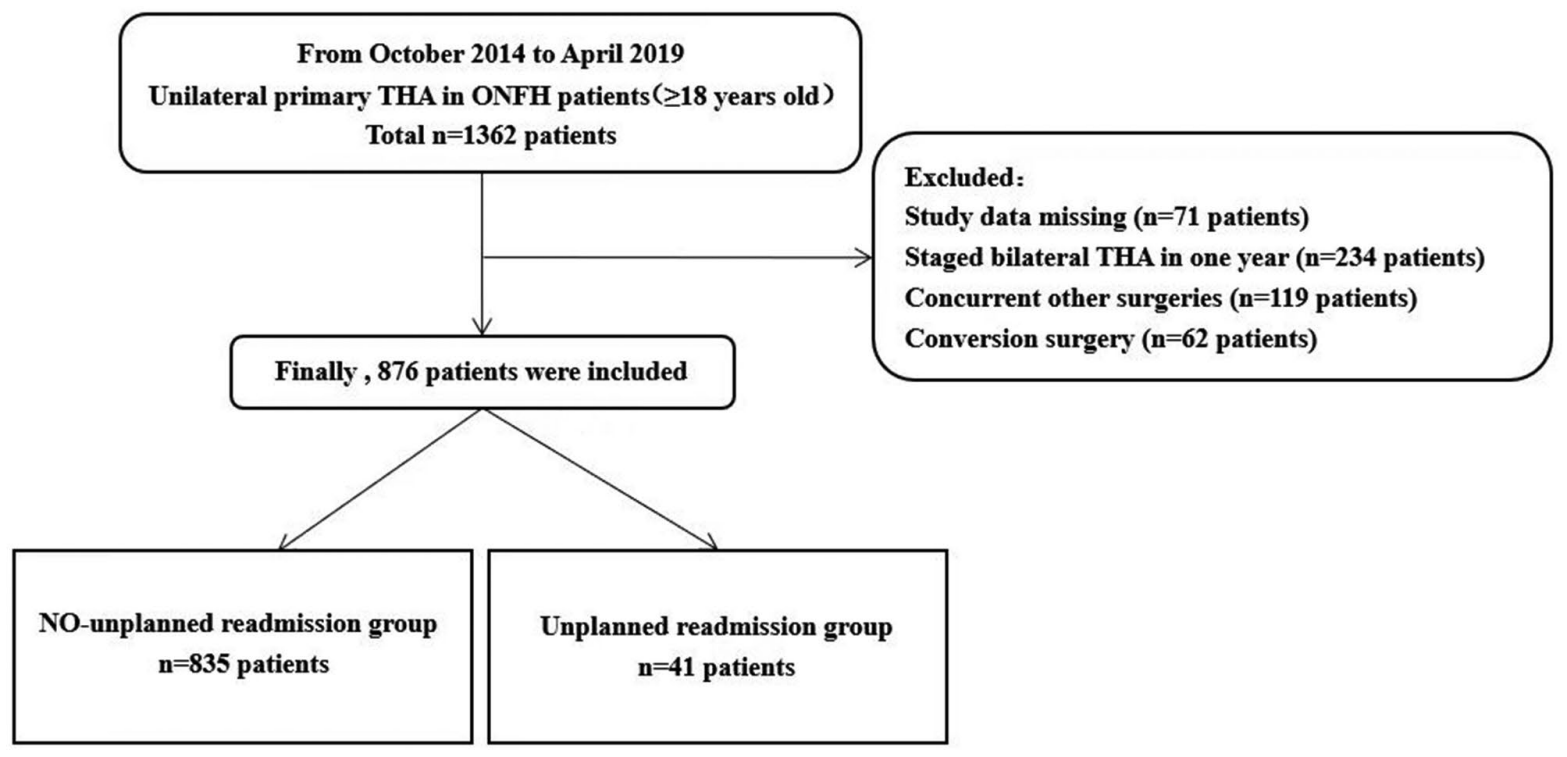
Sample selection flow chart

We retrospectively investigated hospitalized patients 18 years or older who underwent primary unilateral THA for ONFH at the Third Hospital of Hebei Medical University from October 2014 to April 2019. Patients were not considered in the study when: (1) incomplete study data; (2) staged THA in one year for bilateral ONFH; (3) unilateral THA with other surgeries simultaneously; (4) conversion surgery after other hip surgery. (Figure. 1) This was done for two reasons: (1) most THAs are primary unilateral THAs, of more clinical interest; and (2) to keep the good population homogeneous and allow easy interpretation of results.

All data for this cross-sectional study were retrospectively collected from the Surgical Site Infection in orthopedic surgery (SSIOS). The SSIOS database is a prospectively manually maintained database of all data on hospitalized patients who experience orthopedic surgeries at the Third Hospital of Hebei Medical University; data are collected manually by 230 standardized trained investigators and updated annually; the database has provided data support in many previous studies [16–18]. The data in this study were reviewed independently again by three investigators before this research, and any discrepancies were resolved by re-examination. Therefore, the data analyzed in this study can be considered of high quality and precision.

Patient demographic information included sex (male or female), age, residence (rural or urban), height, and weight. BMI was calculated from height in meters and weight in kilograms and divided into 5 groups according to Chinese reference criteria: underweight, < 18.5; normal, 18.5–23.9; overweight, 24-27.9; obesity, 28-31.9; morbid obesity, 32 and above. Smoking, alcohol consumption, history of hip trauma, comorbid conditions, history of food or drug allergy, and any previous surgical history were asked and recorded. The selection of comorbidities was based on the specific diseases that the Charlson comorbidity index was determined with, as well as other diseases proven to be complications of THA [19, 20]. Hypertension, diabetes mellitus, myocardial infarction, chronic lung disease, chronic liver disease, cerebrovascular disease, peripheral vascular signs, and oncologic disease were ultimately included. The preoperative diagnosis was recorded and the stage of ONFH was determined by imaging. The ONFH stage depends on the Association Research Circulation Osseous (ARCO) staging system [4, 21]. We use the ASA index to assess the patient’s physical condition and surgical risk. Surgical information collected included the side of surgery, the anesthesia method, the duration of surgery, intraoperative bleeding, and intraoperative blood transfusion. The duration of surgery was calculated from the time of the skin incision to the time of the skin closure. Length of stay (LOS) was also collected and recorded, which we defined as the number of days from the date of admission to the date of discharge.

### Surgical procedures

The procedures were performed by a posterolateral approach and combined with spinal-epidural analgesia or general anesthesia, as determined by the anesthesiologist on the side. A curved incision of approximately 10 cm was made through the posterior and lateral approaches to the hip joint, and the skin, subcutaneous tissue, and fascia were incised in sequence. The lower limb was straightened and rotated inwards to expose the stop of the external rotator muscle group behind the greater trochanter and the joint capsule inside. The joint capsule was incised, and the hip was then dislocated by inward tucking and internal rotation. The femoral head was amputated approximately 1.5 cm above the lesser trochanter. The joint capsule and its surrounding synovial tissue were removed along the acetabular rim, and the acetabulum was gradually expanded and deepened. After the acetabular fitting successfully, a prosthesis was placed. The affected limb was kept in knee flexion, hip flexion and internal rotation. The femoral stem prosthesis and femoral head prosthesis were installed, and the joint was repositioned. The trauma cavity was irrigated and cleaned, and the incision was sutured layer by layer.

### Treatment of patients

A strict perioperative strategy was implemented when patients were admitted to the hospital. All procedures were performed by surgeons with specialized training in a single institution. In all patients, tranexamic acid was administered in a dose of 1 g in one hour before surgery. Uncemented implants were used in all surgeries. Intraoperative and postoperative antibiotics such as cefazolin or clindamycin were administered prophylactically. Anticoagulation prophylaxis consisted of low molecular weight heparin (LMWH) beginning the night of surgery and continuing for 30 days postoperatively together. Patients were encouraged to walk down to the floor and carry out activities, including crutches, partial weight-bearing, and strengthening exercises for abductor muscles. The medical staff instructed patients to review at 1, 3, 6, and 12 months after discharge.

### Unplanned readmission collection

We identified readmissions by reviewing hospital electronic cases and telephone follow-ups. Unplanned readmission within one year was defined as readmission for the same or related unexpected clinical events within 12 months after index discharge. If the patients were readmitted within one year, we recorded the causes and time in detail. Scheduled admissions, such as reexaminations or other planned procedures, were excluded. When multiple readmissions occurred within one year, only the first unplanned readmission was included in the analysis.

### Statistical analysis

Unplanned readmission rates were calculated by dividing the number of readmitted patients by the total number of patients. The 30-day, 90-day, and 1-year readmission rates were calculated, respectively.

Univariate analysis was performed on the study variables. When the continuous variables were normally distributed, the Student-t test was used, which was expressed as mean ± standard deviation. The Mann Whitney-u test was used for the nonnormal distribution, which was expressed by median and quartile. Categorical variables were expressed as numbers and percentages (%), with Chi-square or Fisher’s exact test as appropriate. The Hosmer–Lemeshow test was used to examine the goodness-of-fit of this model, and P > 0.05 indicated an acceptable fitness. In the result, variables whose value of P < 0.05 were considered independent risk factors. OR values and 95% confidence intervals (CI) were calculated for each correlation. All statistical analyses were conducted with the Statistical Package for Social Sciences (SPSS) software (version 26.0, Chicago, USA).

## Results

### Characteristics of the study patients

In general, 876 patients were included in this study. Of them, 529 were male and 347 were female, with a mean age of 54.47 years (range 19 to 86 years). In total, there were 543 cases of unilateral femoral head necrosis and 333 cases of bilateral femoral head necrosis in the first admission diagnosis. According to the ARCO staging system, 383 people had ARCO stage III and 493 people had ARCO stage IV on the operation side. Finally, 409 cases underwent surgery on the left side and 467 cases on the right.

### Characteristics of unplanned readmission

**Fig. 2:**
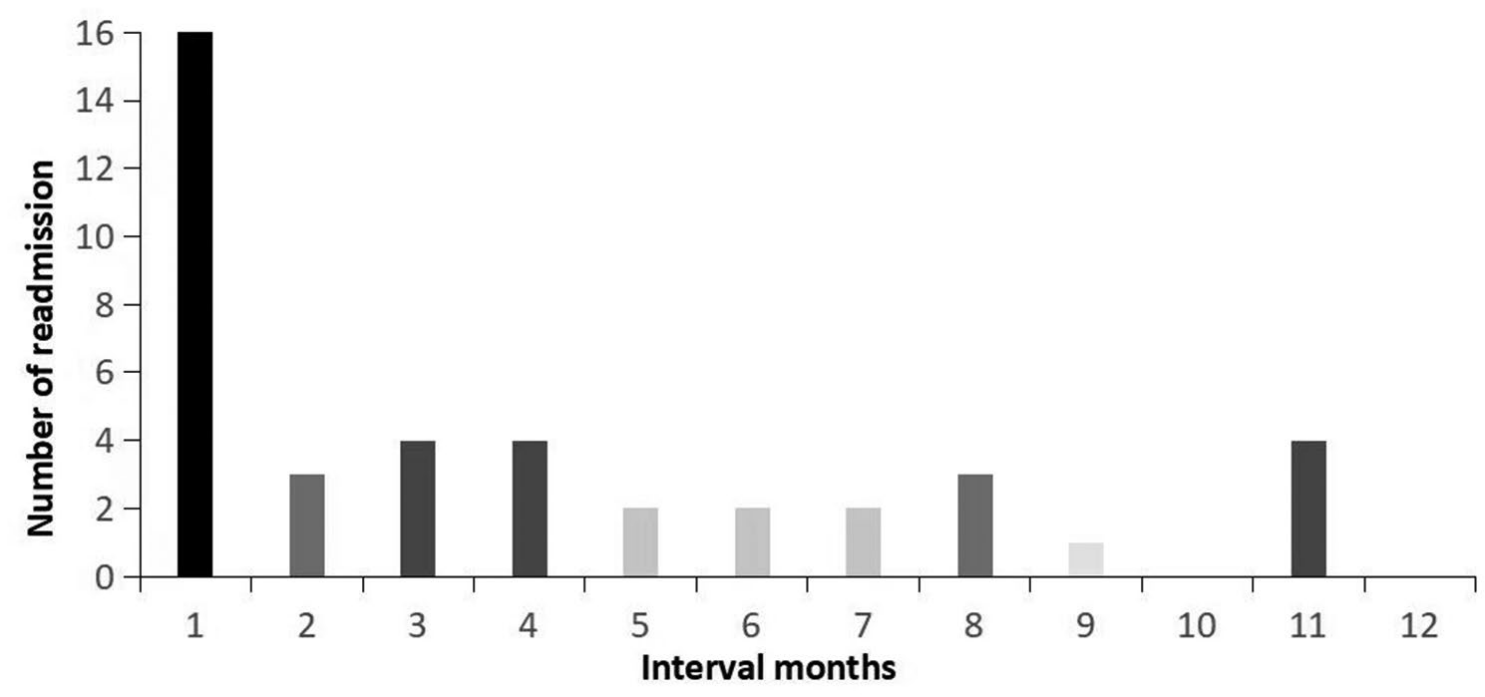
Discharge-readmission month distribution chart

According to the follow-up results, 2 patients after discharge died within one year. One died of cancer, and the other one had a traffic accident, which were both irrelevant to THA. Finally, we determined 41 ONFH patients experiencing unplanned readmission after THA within one year, with readmission rates of 1.83% for 30 days, 2.63% for 90 days, and 4.68% for 1 year. Figure 2 shows the interval time from discharge to readmission, and it can be seen that the largest number of readmission events occur in the first month (39.02%, 16/41). The median time from the last discharge to readmission was 77 days, and the median length of stay for rehospitalization was 8 days.

Table 1 shows the reasons for unplanned readmissions. The account for unplanned readmissions within one year was due to arthroplasty-related causes, 78.0% (32/41); medical-related causes, 22.0% (9/41). Arthroplasty-related causes (in descending order of frequency) included prosthesis dislocation (36.6%), pain (12.2%), deep vein thrombosis (9.8%), periprosthetic fracture (7.3%), incision nonunion (4.9%), hematoma (2.4%), incision skin necrosis (2.4%), and surgical site infection (2.4%). Medical-related causes (in descending order of frequency) included myocardial infarction (4.9%), acute cerebral infarction (4.9%), acute cystitis (4.9%), angina (2.4%) urinary incontinence (2.4%), and inflammatory myopathy (2.4%). Arthroplasty-related conditions were the leading cause of unplanned readmissions, and the most common cause was the dislocation of the prosthesis.

**Table 1:** Causes of unplanned readmission

Cause	0—1 year n (%)	≤ 30 days n (%)	31 to 90 days n (%)	91 to 365 days n (%)
**Surgical related**	32 (78.0)	14 (34.1)	6 (14.6)	12 (29.3)
Dislocation	15 (36.6)	7 (17.1)	3 (7.3)	5 (12.2)
Pain	5 (12.2)	1 (2.4)	1 (2.4)	3 (7.3)
Deep venous thrombosis	4 (9.8)	2 (4.9)	0 (0.0)	2 (4.9)
Periprosthetic fracture	3 (7.3)	1 (2.4)	1 (2.4)	1 (2.4)
Nonunion of incision	2 (4.9)	2 (4.9)	0 (0.0)	0 (0.0)
Hematoma	1 (2.4)	0 (0.0)	0 (0.0)	1 (2.4)
Incision skin necrosis	1 (2.4)	1 (2.4)	0 (0.0)	0 (0.0)
Surgical site infection	1 (2.4)	0 (0.0)	1 (2.4)	0 (0.0)
**Medical related**	9 (22.0)	2 (4.9)	1 (2.4)	6 (14.6)
Myocardial infarction	2 (4.9)	0 (0.0)	0 (0.0)	2 (4.9)
Acute cerebral infarction	2 (4.9)	0 (0.0)	0 (0.0)	2 (4.9)
Acute cystitis	2 (4.9)	1 (2.4)	0 (0.0)	1 (2.4)
Angina	1 (2.4)	1 (2.4)	0 (0.0)	0 (0.0)
Urinary incontinence	1 (2.4)	0 (0.0)	1 (2.4)	0 (0.0)
Inflammatory myopathies	1 (2.4)	0 (0.0)	0 (0.0)	1 (2.4)
**Total**	41 (100.00)	16 (39.0)	7 (17.1)	18 (43.9)

Table 2 presents the distributions of 41 cases and 835 controls. Cases with age > 60 years (70.7%vs34.7%, P < 0.01), urban residence (41.5%vs20.7%, P < 0.01), having cardiovascular disease (14.6%vs4.6%, P = 0.012), having cerebrovascular disease (17.1%vs4.8%, P < 0.01), ARCO stage IV (75.6% vs55.4%, P = 0.026) and smoking (65.9% vs. 48.7%, P = 0.032) had higher rates than those who were not readmitted. The distribution of other characteristics was not statistically significant (P > 0.05).Traumatic experience of hip.

**Table 2:** Comparison of patient characteristics between the two groups

Variables	Non-unplanned readmission	Unplanned readmission	P-value
**Total**	835	41	
**Age (> 60 years)**	290 (34.7%)	29 (70.7%)	<0.01*
**Gender**			0.565
Male	506 (60.1%)	23 (56.1%)	
Female	329 (39.9%)	18 (43.9%)	
**BMI (kg/m** ^**2**^ **)**			0.109
28–32	147 (17.6%)	10 (24.4%)	
>32	37 (4.4%)	4 (9.8%)	
**Place of residence**			<0.01*
Rural	662 (79.3%)	24 (58.5%)	
Urban	173 (20.7%)	17 (41.5%)	
**Cigarette smoking**	407 (48.7%)	27 (65.9%)	0.032*
**Alcohol consumption**	94 (11.3%)	5 (12.2%)	0.853
**History of hip trauma**	28 (3.4%)	4 (9.8%)	0.088
**Comorbidity**			
Hypertension	227 (27.2%)	15 (36.6%)	0.189
Diabetes mellitus	70 (8.4%)	7 (17.1%)	0.102
Cardiovascular disease	38 (4.6%)	6 (14.6%)	**0.012**
Cerebrovascular disease	40 (4.8%)	7 (17.1%)	<0.01*
Chronic pulmonary disease	12 (1.4%)	1 (2.4%)	0.281
Chronic liver disease	32 (3.8%)	3 (7.3%)	0.481
Peripheral vessels sign	16 (1.9%)	2 (4.9%)	0.458
Tumor	13 (1.6%)	0 (0.0%)	0.886
**Allergic history**	136 (16.3%)	8 (19.5%)	0.586
**History of surgery**	289 (34.6%)	14 (34.1%)	0.951
**Admitting diagnosis**			0.237
Unilateral femoral head necrosis	514 (61.6%)	29 (70.7%)	
Bilateral femoral head necrosis	321 (38.4%)	12 (29.3%)	
**ARCO stage**			0.026*
III	372 (44.6%)	11 (24.4%)	
IV	463 (55.4%)	30 (75.6%)	
**ASA score (≥ 3)**	109 (13.1%)	7 (17.1%)	0.459
**Operation side**			0.964
Left	390 (46.7%)	19 (46.3%)	
Right	445 (53.3%)	22 (53.7%)	
**Anesthesia method**			0.870
Combined spinal anesthesia	275 (32.9%)	13 (31.7%)	
General anesthesia	560 (67.1%)	28 (68.3%)	
**Operation time (> 120 min)**	196 (23.5%)	9 (22.0%)	0.822
**Operative blood loss (≥ 400 ml)**	266 (31.9%)	18 (43.9%)	0.108
**Operative blood transfusion**	63 (7.5%)	4 (9.8%)	0.827
**LOS (> 14 days)**	104 (12.5%)	5 (12.2%)	0.961

*Significant variables

### Risk of unplanned readmission

Table 3 examines the OR of the independent risk factors for unplanned readmission through multivariate regression analysis. Risk factors associated with increased risk of unplanned readmission adjustment were age (> 60 years, OR = 3.111, 95%CI = 1.591–6.084, P = 0.001), residence (urban, OR = 3.079, 95%CI = 1.555–6.096, P = 0.001), ARCO stage (stage IV, OR = 2.316, 95%CI = 1.114–4.817, P = 0.025), smoking (OR = 2.166, 95%CI = 1.063–4.412, P = 0.033). There were no significant differences in other factors (P > 0.05).

**Table 3:** Variables tested for multivariate analysis

Variables	P value	Odds ratio	95% CI
Age (> 60 years)*	**0.001**	**3.111**	**1.591–6.084**
Residence (urban)*	**0.001**	**3.079**	**1.555–6.096**
ARCO level (IV)*	**0.025**	**2.316**	**1.114–4.817**
Smoking*	**0.033**	**2.166**	**1.063–4.412**
Cerebrovascular disease	0.081	3.122	0.869–11.211
Cardiovascular disease	0.746	1.254	0.319–4.936

CI: cnfidential interval

*Significant variables

## Discussion

To our knowledge, this was the first study to focus on unplanned readmission after THA in patients with ONFH. According to follow-up results, 41 patients (4.68%) had at least one readmission within one year. The readmission rate was 1.83% in 30 days 2.63% in 90 days, and 4.68% in 1 year. At every time point studied, the dislocation of the prosthesis was always the most common cause. Age > 60 years, smoking, ARCO stage IV, and urban residence settings were considered independent risk factors for unplanned readmission.

In this study, the prevalence of unplanned readmission occurred at 1.83% in 30 days and 2.63% in 90 days, which was significantly lower than most previous reports (5.1%, 6.11% in 30 days, and 9.6%, 13.1% in 90 days) [6, 13–15]. Only the study by Lovecchio et al., reporting a 30-day readmission rate of 1.6%, was close to ours [5]. One potential reason for this variation is the difference in patient management interventions between institutions. ONFH patients were generally in poor health and had more comorbidities. Using a database from California, Stavrakis et al. suggested that preoperative and postoperative optimization of medical issues in ONFH patients could effectively limit readmissions [14]. Lovecchio et al. also recommend that surgeons take appropriate perioperative measures to reduce readmissions, and their low readmission rate well supported this opinion [5]. Similarly to their suggestions, our hospital has always implemented a strict perioperative strategy. Surgeons would perform detailed examinations and assessments of the patients before surgery. For those with comorbidities, timely physical adjustments would be made, as well as close post-operative monitoring and preventive measures. Although the complex management process may increase initial hospital days, it effectively reduced hospital readmissions for medical-related problems. In the first days after discharge, only a small proportion of readmissions were medical-related (12.5% at 30 days and 13.0% at 90 days), compared with nearly half in some studies [22, 23].

Prosthesis dislocation is the most common cause of readmission and occurs frequently in all periods of the year. Indeed, prosthesis dislocation occurs easily in patients with ONFH. Yang et al. studied complications within 2 years after THA and reported that patients with ONFH had a significantly higher dislocation rate for nearly all time points studied than those without ONFH [24]. Yoshimoto et al. retrospectively reviewed revision THA in patients between 1998 and 2013 and found that ONFH was a risk factor for prosthesis dislocation after THA. In their study, patients with ONFH were 7.71 times more likely to suffer a prosthesis dislocation [25]. The strong connection between ONFH and prosthesis dislocation is believed to be the cause of ONFH itself. ONFH patients generally drink alcohol or use corticosteroids. Alcohol consumption has a seriously detrimental effect on cognitive status and results in poor patient compliance with preventive measures to avoid dislocation [24, 25]. In the early days after THA, patients were encouraged to walk with crutches and partially weight bearing. However, if patients overexerted themselves under the interference of alcohol, the immature scar provided only weak mechanical support for joint stability, highly susceptible to dislocation [26] In our study, almost half of the prosthesis dislocations occurred within the first 30 days. These patients required special interventions, including improved patient education, detailed discharge planning, and regular follow-up communication. For patients using corticosteroids, the drug would cause secondary changes in the soft tissues around the hip joint, leading to prosthesis dislocation [25]. The lateral approach, larger femoral head sizes, and double action cups may be helpful [27].

Age has been widely reported as a risk factor for readmission in patients after THA. In a retrospective study of 12,030 patients, Paxton et al. found that each additional year of patient age was associated with a 3% higher likelihood of readmission at 30 days [28]. In a study that focused on the effect of advanced age on THA, Fang et al. also found similar evidence. They identified that patients were more likely to experience postoperative complications with increasing age and the reported rates of all-cause 30-day readmissions increased from 1.9% (51–60 years) to 3.8% (61–70 years), approximately double [29]. Due to the coexisting conditions associated with ONFH, elderly ONFH patients may face higher risks. But Murphy et al. pointed out that although increasing age was associated with complications within 12 months after THA, the elderly could still obtain equivalent benefits from THA as those younger adults when risks were handled well [30]. For the elderly who require THA, surgeons must be aware of their worse health situation and take measures actively, such as maximizing their health status preoperatively and closely monitoring them during postoperative hospitalization.

Smoking, a common health and economic concernm, was believed to have a significant negative effect on THA. Nicotine compromises the distribution of peripheral blood and oxygen, which can impair all phases soft tissue and skin healing. It also has deleterious effects on bone metabolism and vital organs [31]. Several large retrospective studies have shown that smoking significantly increases the risk of all postoperative complications, including serious medical diseases, prosthetic joint infection, wound problems, and early revision after THA [32, 33]. In a propensity score-matched analysis, Sahota et al. found that smokers had almost twice readmission rate than non-smokers(4.3% vs. 2.2%) [34]. In our multivariate logistic analysis model, smoking increased the risk of readmission up to 2.166 times. Fortunately, smoking is a controllable variable. Smoking cessation significantly reduces post-operative morbidity. The surgeon can clearly inform the patient that quitting significantly reduces the risk of readmission, providing a compelling argument for the patient to consider quitting. It’s also worth noting that long-term, intensive smoking cessation programmes and regular face-to-face contact can produce better results than short-term cessation alone [31–34].

We connected the ARCO stage with unplanned readmissions of ONFH patients after THA for the first time. ARCO stage IV, the final stage of ONFH, left patients in a state of more prolonged immobility of the lower limb and hypercoagulable blood, which more easily induced various chronic diseases such as cardiovascular diseases and obesity [35, 36]. Furthermore, ARCO IV patients show a narrowing of the joint space, acetabular changes, and destruction, which increases the difficulty of the operation, such as removing the diseased femoral head, cleaning up the malformed acetabular medial wall and periacetabular osteophytes, releasing the surrounding contracted muscles, and precise placement of acetabular prostheses [27]. We also find that the relationship between the ARCO stage and unplanned readmission may be related to the negative emotions caused by ONFH. ARCO stage IV patients are exposed to negative emotions from pain and immobility caused by ONFH over a longer time. In a questionnaire-based analysis of 216 individuals, Chen et al. indicated that, compared to the early stage, advanced stages of ONFH were likely to suffer mental disorders, which play a negative role in the long-term outcomes of patients undergoing total hip arthroplasty, including higher rates of abnormal discharge and more surgical complications [37]. For patients with ARCO stage IV, hospitals should notice their mental health and provide appropriate psychological counseling while optimizing patient physical conditions.

The difference in readmission between urban and rural areas can be explained by economic income, medical resources, and education level. Urban patients have higher economic incomes and better creature comforts, but these also lead to more chronic diseases such as hypertension and obesity [38–40]. In addition, urban patients have excellent medical resources and pay more attention to their health. If they are sick or if a problem is identified during postoperative follow-up, they will actively seek treatment. Due to financial restrictions or inconvenience to hospitals, some rural patients may not have regular check-ups and hidden problems may go undetected. Another point worth noting was that, although we used telephone follow-ups to minimize lost readmissions due to rural patients seeking medical care nearby, we still cannot avoid differences caused by education level in the perception of readmissions between rural and urban patients, i.e., rural patients may consider some readmissions as irrelevant to THA, resulting in missed readmissions.

Our study has several strengths. To our knowledge, no one has previously focused on unplanned readmission after THA in ONFH patients. We developed a study on this topic for the first time and provided new references and recommendations. In addition, when we designed the study, we chose a relatively long study period of one year to include more meaningful readmission events. And we try our best not to miss any readmission events. When we counted readmitted patients, we not only inquired about the cases from our hospital electronic case system but also conducted telephone follow-ups. Thus, we can also clearly show the specific reasons and times for the readmissions. In addition, we included enough covariates to reduce confounding bias as much as possible, and some of them were of great meaning and never considered, such as the ARCO stage. However, there were still several limitations in the present study. First, since the posterolateral approach was used in all cases, our findings might lack universality for patients who used a different approach. In addition, we could not determine the connection between each underlying reason for ONFH and readmission, because some patients were uncertain whether they had used corticosteroids and many reasons had superposition. Finally, patient-specific covariates (such as smoking, comorbid conditions, etc.) mainly relied on the patient’s self-report. The accuracy of the information depended on the patient’s knowledge of their medical conditions.

## Conclusions

In summary, the readmission rate was 1.83% in 30 days, 2.63% in 90 days, and 4.68% in 1 year. Dislocation was the most common cause for all time points studied. Age > 60 years, smoking, ARCO stage IV, and urban residence were the most influential risk factors for readmission within one year. Therefore, interventions aimed at one-year readmissions should pay more attention to preventing prosthesis dislocation, especially in the early days after surgery. In addition, we recommend the introduction of a strict smoking cessation program prior to surgery and the development of comprehensive management strategies, especially for the elderly and end-stage ONFH patients.

## Data Availability

All the data used are available from the corresponding author on motivated requests.

## References

[1] Liu F, Wang W, Yang L, Wang B, Wang J, Chai W, Zhao D (2017). An epidemiological study of etiology and clinical characteristics in patients with nontraumatic osteonecrosis of the femoral head. J Res Med Sci.

[2] Cooper C, Steinbuch M, Stevenson R, Miday R, Watts NB (2010). The epidemiology of osteonecrosis: findings from the GPRD and THIN databases in the UK. Osteoporos Int.

[3] Learmonth ID, Young C, Rorabeck C (2007). The operation of the century: total hip replacement. Lancet.

[4] Zhao D, Zhang F, Wang B, Liu B, Li L, Kim SY, Goodman SB, Hernigou P, Cui Q, Lineaweaver WC (2020). Guidelines for clinical diagnosis and treatment of osteonecrosis of the femoral head in adults (2019 version). J Orthop Translat.

[5] Lovecchio FC, Manalo JP, Demzik A, Sahota S, Beal M, Manning D (2017). Avascular necrosis is Associated with increased transfusions and readmission following primary total hip arthroplasty. Orthopedics.

[6] Singh JA, Chen J, Inacio MCS, Namba RS, Paxton EW. An underlying diagnosis of osteonecrosis of bone is associated with worse outcomes than osteoarthritis after total hip arthroplasty. BMC Musculoskelet Disord 2017, 18(1). 10.1186/s12891-016-1385-0 PMC5223478 28068972

[7] Schairer WW, Sing DC, Vail TP, Bozic KJ (2013). Causes and frequency of Unplanned Hospital Readmission after total hip arthroplasty. Clin Orthop Relat Research®.

[8] Slullitel PA, Estefan M, Ramírez-Serrudo WM, Comba FM, Zanotti G, Piccaluga F, Buttaro MA (2018). Re-admissions treble the risk of late mortality after primary total hip arthroplasty. Int Orthop.

[9] Ali AM, Loeffler MD, Aylin P, Bottle A. Factors Associated with 30-Day readmission after primary total hip arthroplasty. JAMA Surg 2017, 152(12). 10.1001/jamasurg.2017.3949 PMC5831438 28979994

[10] Healy WL, Iorio R, Clair AJ, Pellegrini VD, Della Valle CJ, Berend KR (2016). Complications of total hip arthroplasty: standardized list, definitions, and Stratification developed by the Hip Society. Clin Orthop Relat Res.

[11] Cui L, Zhuang Q, Lin J, Jin J, Zhang K, Cao L, Lin J, Yan S, Guo W, He W (2015). Multicentric epidemiologic study on six thousand three hundred and ninety five cases of femoral head osteonecrosis in China. Int Orthop.

[12] Pabinger C, Lothaller H, Portner N, Geissler A (2018). Projections of hip arthroplasty in OECD countries up to 2050. HIP Int.

[13] Sax OC, Pervaiz SS, Douglas SJ, Remily EA, Mont MA, Delanois RE (2021). Osteoarthritis and osteonecrosis in total hip arthroplasty: 90-Day postoperative costs and outcomes. J Arthroplast.

[14] Stavrakis AI, SooHoo NF, Lieberman JR (2015). A comparison of the incidence of Complications following total hip arthroplasty in patients with or without osteonecrosis. J Arthroplast.

[15] Sodhi N, Anis HK, Coste M, Piuzzi NS, Jones LC, Mont MA (2020). Thirty-day Complications in osteonecrosis patients following total hip arthroplasty. J Arthroplast.

[16] Wang Z, Zheng Z, Ye P, Tian S, Zhu Y, Chen W, Hou Z, Zhang Q, Zhang Y (2022). Treatment of tibial plateau fractures: a comparison of two different operation strategies with medium-term follow up. J Orthop Translation.

[17] Zhang K, Zhu Y, Tian Y, Tian M, Li X, Zhang Y. Role of a new age-adjusted D-dimer cutoff value for preoperative deep venous Thrombosis exclusion in elderly patients with hip fractures. J Orthop Surg Res 2021, 16(1). 10.1186/s13018-021-02801-y PMC8557539 34717681

[18] Wang Y, Deng X, Wang Z, Zhu Y, Chen W, Zhang Y (2021). Total hip arthroplasty or hemiarthroplasty for femoral neck fractures in elderly patients with neuromuscular imbalance. Aging Clin Exp Res.

[19] Charlson ME, Carrozzino D, Guidi J, Patierno C (2022). Charlson Comorbidity Index: a critical review of Clinimetric Properties. Psychother Psychosom.

[20] Sadoghi P, Liebensteiner M, Agreiter M, Leithner A, Böhler N, Labek G (2013). Revision Surgery after total joint arthroplasty: a complication-based analysis using Worldwide Arthroplasty registers. J Arthroplast.

[21] Cao H, Guan H, Lai Y, Qin L, Wang X (2016). Review of various treatment options and potential therapies for osteonecrosis of the femoral head. J Orthop Translat.

[22] Saucedo JM, Marecek GS, Wanke TR, Lee J, Stulberg SD, Puri L (2014). Understanding Readmission after primary total hip and knee arthroplasty: who’s at risk?. J Arthroplast.

[23] Vorhies JS, Wang Y, Herndon J, Maloney WJ, Huddleston JI (2011). Readmission and length of stay after total hip arthroplasty in a National Medicare Sample. J Arthroplast.

[24] Yang S, Halim AY, Werner BC, Gwathmey FW, Cui Q (2015). Does Osteonecrosis of the femoral head increase Surgical and Medical Complication Rates after total hip arthroplasty? A Comprehensive Analysis in the United States. HIP Int.

[25] Yoshimoto K, Nakashima Y, Yamamoto T, Fukushi J-i, Motomura G, Ohishi M, Hamai S, Iwamoto Y (2015). Dislocation and its recurrence after revision total hip arthroplasty. Int Orthop.

[26] Kostewicz M, Szczęsny G, Tomaszewski W, Małdyk P. Narrative review of the mechanism of hip prosthesis dislocation and methods to reduce the risk of dislocation. Med Sci Monit 2022, 28. 10.12659/MSM.935665 PMC9215209 35715941

[27] Hailer NP, Weiss RJ, Stark A, Kärrholm J (2012). The risk of revision due to dislocation after total hip arthroplasty depends on surgical approach, femoral head size, sex, and primary diagnosis. Acta Orthop.

[28] Paxton EW, Inacio MCS, Singh JA, Love R, Bini SA, Namba RS (2015). Are there modifiable risk factors for Hospital Readmission after total hip arthroplasty in a US Healthcare System?. Clin Orthop Relat Res.

[29] Fang M, Noiseux N, Linson E, Cram P (2015). The Effect of advancing age on total joint replacement outcomes. Geriatric Orthop Surg Rehabilitation.

[30] Murphy BPS, Dowsey MM, Spelman T, Choong PFM (2018). What is the impact of advancing age on the outcomes of total hip arthroplasty?. J Arthroplast.

[31] Singh JA (2011). Smoking and outcomes after knee and hip arthroplasty: a systematic review. J Rhuematol.

[32] Debbi EM, Rajaee SS, Spitzer AI, Paiement GD (2019). Smoking and total hip arthroplasty: increased inpatient Complications, costs, and length of Stay. J Arthroplast.

[33] Matharu GS, Mouchti S, Twigg S, Delmestri A, Murray DW, Judge A, Pandit HG (2019). The effect of Smoking on outcomes following primary total hip and knee arthroplasty: a population-based cohort study of 117,024 patients. Acta Orthop.

[34] Sahota S, Lovecchio F, Harold RE, Beal MD, Manning DW (2018). The Effect of Smoking on thirty-day postoperative Complications after total joint arthroplasty: a propensity score-matched analysis. J Arthroplast.

[35] Tan B, Li W, Zeng P, Guo H, Huang Z, Fu F, Gao H, Wang R, Chen W (2020). Epidemiological study based on China Osteonecrosis of the femoral Head Database. Orthop Surg.

[36] Terra MF, Pedrosa DG, Zoppi CC, Werneck CC, Vicente CP (2019). Physical exercises decreases thrombus and neointima formation in atherosclerotic mice. Thromb Res.

[37] Matsuoka Y, Chen S-B, Hu H, Gao Y-S, He H-Y, Jin D-X, Zhang C-Q. Prevalence of clinical anxiety, Clinical Depression and Associated Risk factors in Chinese young and middle-aged patients with osteonecrosis of the femoral head. PLoS ONE 2015, 10(3). 10.1371/journal.pone.0120234 PMC4366265 25789850

[38] Yu Q, Lin S, Wu J (2021). Hypertension prevalence Rates among Urban and Rural older adults of China, 1991–2015: a standardization and decomposition analysis. Front Public Health.

[39] Xiaohui H (2008). Urban-rural disparity of overweight, Hypertension, undiagnosed Hypertension, and untreated Hypertension in China. Asia Pac J Public Health.

[40] Kun P, Liu Y, Pei X, Luo H (2013). Regional and urban-rural disparities in prevalence of over-weight among old people in China: evidence from four Chinese provinces. J Nutr Health Aging.

